# Translating psychosocial safety climate (PSC) into real-world practice: two PSC intervention case studies

**DOI:** 10.1093/joccuh/uiae051

**Published:** 2024-09-05

**Authors:** May Young Loh, Maureen F Dollard, Sarven S McLinton, Paula Brough

**Affiliations:** Psychosocial Safety Climate Global Observatory, University of South Australia, Adelaide, Australia; Psychosocial Safety Climate Global Observatory, University of South Australia, Adelaide, Australia; Psychosocial Safety Climate Global Observatory, University of South Australia, Adelaide, Australia; Centre for Work, Organisation and Wellbeing, Griffith University, Nathan, Australia

**Keywords:** psychosocial safety climate, occupational health intervention, psychological health, psychological well-being, case studies, job redesign

## Abstract

Objectives: Translating research into practice is often a goal for evidence-based organizational researchers to help improve workplace conditions and worker well-being. Improving worker well-being can be achieved by using empirical evidence to inform organizational interventions. However, despite the well-established intervention literature, practitioners appear not to appreciate fully how research findings can inform real-world practice. Using our understanding about workplace safety and health issues, we proposed that employers themselves could undertake interventions that focus on building psychosocial safety climate (PSC), an essential organizational climate that protects and promotes the psychological well-being of workers.

Methods: Here we present 2 case studies to illustrate strategies that improve psychosocial safety and to increase our understanding about how interventions help improve PSC over time. Case Study 1 was conducted in an Australian public organization and Case Study 2 was in an international private organization. We collected survey data using the PSC-12 scale, to assess the level of PSC of the organization before and after the intervention, and details of the intervention and other initiatives for promoting employees’ psychological health.

Results: Our evaluation supported the proposition that interventions that combine organizational-level and individual-level (and the interface between the two) approaches with a focus on the core elements of PSC (such as commitment, priority, communication, and participation) improve an organization’s PSC over time.

Conclusions: The research not only elucidates important practical implications for organizations trialing new psychosocial safety initiatives, but also makes an important contribution to theory in work stress intervention on best practice and principles to build a psychologically healthy work context.

## Teaser

Key points

- **What is already known on this topic**: Organizational stress interventions are important to ensure workers’ health and safety. Useful guidelines are available to help design, execute, revise, and evaluate workplace interventions, particularly in the globalized era and large multinational companies. However, challenges remain as interventions are often multifactorial, multifaceted, and involve different parties. Additionally, the gap between theory and practice remains wide. Hence there is a role for academics in expanding literature and understanding the elements required to build knowledge for both practitioners and academia. Detailed guidelines and examples will be provided to assist future development in this topic.
- **What this study adds**: This article provides examples of 2 successful integrated in-house interventions, led by the organization’s workplace champions in collaboration with academics. Activities and processes of the interventions illustrate that a social coordination among the employers, employees (ie, workplace champions), and the academics is useful in protecting and promoting a psychologically healthy workplace.
- **How this study might affect research, practice, or policy:** We contribute to the knowledge of a system approach to work stress intervention and to the development of sustainable and participatory interventions, by applying the PSC principles. A few examples have detailed how organizational internal experts can integrate the elements of PSC into their existing policies and practices, thereby amending and rectifying their risk management system.

## 1. Introduction

Work-related stress has significant negative consequences for employee psychological health and well-being, with flow-on performance and productivity effects, such as increased sickness absence and employee turnover. Stakeholders, such as the policymakers, employers, organizational psychologists, and public health experts, should therefore direct attention toward reducing threats to worker psychological health and promoting decent work.^1^ Scholars have increasingly focused on improving employee well-being through organizational interventions.^2^ However, the wide gap between theory and practice for occupational stress interventions has been widely acknowledged.^3^^‑^^5^ Without theory, the outcome of the intervention is questionable.^6^ The degree to which interventions have created a real and sustainable impact in organizations is also debatable. Nielsen and Brough^6^ identified several important theories underlying the process of participatory interventions, including collaborative job crafting, workers’ sense-making, workplace cultures, and trust. Psychosocial safety climate (PSC) is likely to influence these concepts (job crafting, trust) so we use the PSC theory to frame the process of the intervention. To integrate these ideas, in the current study, we focus on building a positive workplace culture^6^ through PSC theory.^7^

PSC is the organizational climate for employee psychological health. Under PSC theory, a high PSC organization is an organization where employees are confident to voice their problems and have faith that an organization will take a supportive course of action when faced with risks.^8^ PSC is the “cause of causes” of employee psychological health and safety^7^ because it predicts psychosocial risk factors known to affect employee psychological health. High PSC contexts cultivate job conditions with manageable levels of job demands (ie, job aspects that require effort) and enriched job resources (ie, job aspects that help one deal with job demands). Accordingly, high PSC correlates to lower levels of emotional exhaustion, psychological distress, and depression and to higher levels of work engagement, job satisfaction, and performance.^7^ Moreover, PSC is positively related to team job crafting,^9^ potentially an ingredient for intervention successfulness.^10^ As such, PSC is a useful target in the risk management process, particularly in turbulent times, such as organizational restructuring and a global pandemic.^11^ It consists of 4 principles that play important roles in the process of intervention, namely, management priority for psychological health over productivity, management support and commitment, organizational communication, and organizational participation and involvement. Hence focusing on PSC theory to integrate the important elements for an organizational intervention is warranted.

Importantly, espoused policies are not enough to create real change. It is necessary to enact policies and practices to successfully create change in an organization. Similarly, scholars suggest that researchers should convert or incorporate interventions into existing mainstream policies, by integrating the intervention into regular daily operations and create a routine for promoting and preventing work stress.^12^

Hence, how to build PSC is an important question, and interventions addressing this are receiving increased attention in the literature. For example, Dollard and Bailey^11^ successfully found an improvement in PSC during the global pandemic through capacity-building training. Bronkhorst and colleagues^13^ also found that organizational PSC could be improved by having safety-related meetings, discussions, and leadership training. However, a widely noted limitation of occupational stress intervention research is that the benefits arising from the intervention are often short-lived, and markedly decline after researchers leave the participatory organization.^6^ Therefore, one way to create sustainable change in an organization is to involve experts within the organization (ie, workplace champions) to co-design and embed the intervention.

In this article we present 2 organizational case studies that implemented PSC interventions. We outline a collaboration process between the researchers and occupational health and safety experts employed by the organization to create an organization-wide intervention that could embed lasting change in the organization’s policies, practices, and procedures. To better understand the intervention process, we documented all intervention activities in the 2 case studies illustrating the social coordination between researchers, organization, and safety representatives.

### 1.1. Stress management interventions

The literature of workplace intervention has evolved tremendously in the past 20 years, moving from an individual-level focus to an organizational-level and/or multilevel perspective. The literature supports this movement and suggests that quality work stress interventions should involve all parties in organizations (ie, employees, middle-level management, top executives, and other stakeholders)^6^^,^^14^; focus on prevention rather than treatment (ie, proactive rather than reactive)^15^; involve employee participation^6^; increase organizational ownership^16^; and be mindful of the context.^6^ These best practices should be followed throughout the process of designing, implementing, and evaluating an intervention.^2^ The major steps to be followed include conducting a risk assessment, action planning, involving stakeholder participation, and evaluating action plans.

In relation to the content of intervention, scholars have suggested that interventions could target 3 foci: primary (ie, targeting the root cause of stress), secondary (ie, improving skills to cope with stress), and tertiary (ie, treatment after exposure to stress) intervention.^15^ Intervention researchers have highlighted that of these foci, primary intervention is the most effective. As research suggests, the root cause of stress often lies at the top level of an organization, which is why primary stress interventions should focus on changing policies, practices, and procedures.^12^ In a practical sense, a risk assessment involving understanding the gaps in existing psychosocial risk management systems is appropriate. These risk assessments should involve participation from all levels of the organization either directly or indirectly.^6^ All workers should be involved in the discussion and action planning process. However, this is difficult to achieve in larger organizations. Hence, indirect participation could be achieved by engaging with representatives of workers, as is illustrated in our study.

In addition, organizations have a hierarchical nature, and organizational health and safety issues that stem from them have different levels of influence.^6^^,^^14^ A multilevel intervention is therefore highly recommended. LaMontagne et al,^14^ in a review of interventions, found that the most effective focus was on 3 levels: an organization’s physical features, the individuals, and an organization’s interface with individual workers. They argued that the primary, secondary, and tertiary foci of intervention are not mutually exclusive. Therefore, to ensure the greatest influence, researchers should consider a system approach by combining different types of interventions that target the organization, the individual, and their interface. We propose that to improve PSC in an organization, practitioners should deliver interventions that focus on a combination of organizational, interface, and individual levels.

In improving PSC, we focus on implementing and practicing the principles of PSC, which are senior management commitment, senior management priority, organizational communication, and organizational participation. These principles align with the main practices of organizational interventions that are the drivers and enablers of organizational intervention successfulness.^17^ Therefore, when targeting and assessing PSC, we are measuring both a contextual factor and also a process factor,^3^ pertaining to how the intervention is implemented. Intervention success requires management support, good communication, and participation, and these are the core elements of PSC. Targeting PSC for improvement and assessing change in PSC gives information about how effective the intervention is in improving PSC. A PSC intervention is a specifically designed intervention that focuses on how to enact and exert the PSC principles throughout the organization’s structures, job aspects, values and policies.

**Figure 1:**
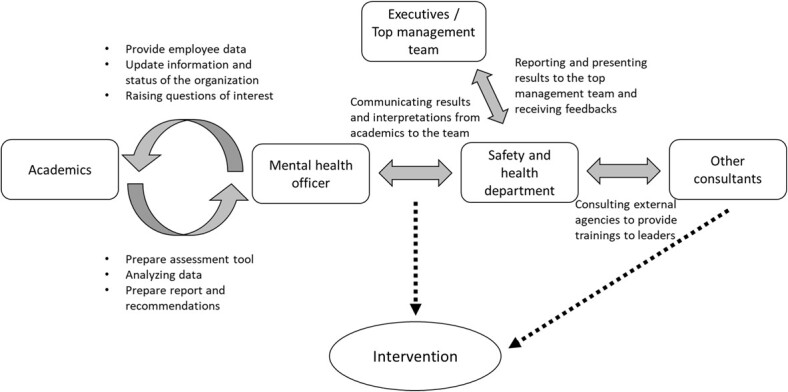
The social coordination process.

This process of a system approach for PSC intervention involves a positively socially coordinated process with a priority on workers’ psychological health.^18^ Social coordination between stakeholders (ie, employers, employees, and regulators/researchers) is formed to oversee the implementation of an intervention. This involves leveraging external and internal organizational resources to achieve a shared goal or vision—in our case, to form a conducive condition for a stronger and higher PSC. Through regular social dialogue, workers, employers, and other parties, such as researchers or regulators, communicate the needs, concerns, and any related demands at workplaces and exchange insights, knowledge, and resources. Social coordination and cooperation and consultation in resolving stress-related issues through diverse changes in organizational policies, procedures, and practices are expected to build PSC. In this article we outline 2 examples of how PSC principles have been upheld in the organization’s activities, programs, protocols, and procedures, which are likely to engender long-term PSC performance.

## 2. Case studies

In the current article we report on 2 case studies that were conducted to understand how PSC could change in an organization over time. The researchers worked with organizational intervention champions^3^ in both case studies (Figure 1), who were either the personnel working in the safety and well-being department or the head of the organization’s well-being department who showed initiatives to improve workplace psychological safety and health. The researchers’ main tasks included providing suitable measurement tools and reports and sharing information and knowledge with the internal practitioners. The researchers were not involved in any intervention processes, including the design and implementation of the intervention. Employees were invited to participate with the survey and risk assessment to express their concern and their evaluation of the working environment. However, researchers followed best practice principles^6^ regarding how to intervene by assisting in a risk assessment, identifying the area of focus, and performing a follow-up evaluation regarding how the intervention influenced the organization’s level of PSC. The employee representatives took responsibility for proposing, designing, and organizing the activities within the organizations.

### 2.1. Case Study 1

#### 2.1.1. Background and context

An anonymous survey was conducted to assess an Australian government organization’s level of PSC. The safety and well-being department informed all employees about the study via email, outlining the study’s aims, privacy protection strategies, and researchers’ contact information. Ethics approval was obtained from a collaborative partner, the University Technology Sydney (ethics protocol ETH193527). A total of 690, 646, and 2136 responses from 2018 (Time 1 [T1]), 2019 (T2), and 2021 (T3), respectively, were included in the analysis. (The large cohort at T3 was due to the integration of the survey into a larger company survey that was collected annually. Surveys at T1 and T2 were sent out separately and had lower response rates.) Due to the global pandemic, the survey was delayed in year 2020. In year 2018, no demographic or other personal related information was collected. In 2019, 37.5% of the respondents were female, median age group was 45-55 years old, and 577 respondents were working full-time. In year 2021, 26% of the respondents were female, 94.7% were working full-time, and median age group was 45-55 years old.

**Table 1:** Activities and programs conducted in Case Studies 1 and 2.

**PSC domain**	**Initiatives of Case Study 1**	**Initiatives of Case Study 2**	**Level of intervention**
Priority	1. An external work safety consultant conducted a risk assessment for the organization to identify any gaps in their systems, policies, and procedures		Organizational
Participation and involvement	2. A survey was conducted to collect employees’ perspectives regarding their existing policies, practices, and procedures	1. A flexible working policy was communicated, and additional days of well-being leave were provided to all staff during the pandemic	Organizational
Commitment and support	3. Executive training (1 session) was provided for 83% of Level 2 and Level 3 managers in June 2018. This training addressed: • PSC, while also suggesting actions regarding specific areas on which each group should focus based on their results throughout the 12 questions and 4 focus areas • psychosocial risk factors and discussion on how to minimize these factors within their work groups	2. A specific intervention was conducted with 7 teams who reported low PSC in 2021. The JD-R model was introduced and action plans were created with a focus on psychosocial factors in each team. The intervention was conducted in partnership with other external, non-university consultants. Action plans were discussed and were in the progress of being implemented at the time of writing of this article	Organizational
Commitment and support	4. Manager training (1 session) was provided for 44% of Level 4 and Level 5 managers in October 2018 (50 leaders), focusing on: • how managers can recognize, respond, refer, and reconnect to mental well-being issues in the workplace • how managers can address a critical capability gap in the population	3. Training was provided to 750 leaders in face-to-face sessions on the topics of PSC and psychosocial risks at work	Organizational/ individual
		4. Fully remote roles were introduced in response to the pandemic, and after the pandemic started to recede, returning to the office was made optional	Organizational/individual
Communication	5. An engagement campaign was created, in which staff could support their well-being across the well-being spectrum		Organizational/individual
Communication	6. The psychological health program was rebranded to reduce stigma	5. Sick leave was rebranded as well-being leave to encourage proactive leave taking and to reduce the stigma	Organizational/individual
Commitment and support	7. The scope of the Employee Assistance Program (EAP) was increased to offer support across the well-being spectrum. The scope includes exercise physiologists, dietitians, legal professionals, financial professionals, psychologists, and counselors		Individual
Communication	8. The engagement program was conducted to introduce the new EAP coaching and support system		Individual
Participation and involvement	9. A safety representative program and coaching training and approach were provided	6. Eighty safety representatives were recruited, and they had manager approval to spend 1-2 h weekly on well-being-related projects. Their role is to drive local well-being projects in each region and office. They have the autonomy and budget to operate within the well-being strategic framework—the integrated model of workplace well-being	Individual
Participation and involvement	10. Lived experience videos for mental health and well-being goals were created		Individual
Priority/communication	11. A mental health awareness event was launched	7. Events and programs were conducted to increase employees’ awareness of psychological health and safety	Individual
Priority/communication	12. An online course on mental well-being was developed	8. An eLearn platform about mental health was released to promote mental health literacy, knowledge of psychosocial factors at work, and skills for supportive conversations in the workplace	Individual
Priority	13. Physical health services were provided (ie, health checks, flu vaccinations, and health promotion campaigns)	9. Physical health services were provided, such as health checks, flu vaccinations, and health promotion campaigns	

Abbreviations: EAP, Employee Assistance Program; JD-R, job demands-resources; PSC, psychosocial safety climate.

#### 2.1.2. Intervention

The organization designed and led a series of stress intervention programs. Table 1 outlines the activities performed from October 2018 to October 2021 highlighting different levels (ie, organization, organization-individual interface, and individual) of intervention. The researchers led a risk assessment at the organizational level, which demonstrated a need to improve leadership skills and provide better training for leaders to manage psychosocial factors. Management commitment and support in tackling psychological health issues contribute to building an effective climate. Commitment includes making prompt decisions and taking actions to solve issues in a specific area affecting workers’ psychological health. For example, when employees approach their supervisor about work overload, a leader should know how to manage the situation by adjusting the workload and/or providing necessary resources to help employees manage the demands. A high level of commitment and support is also reflected through the leaders’ willingness to learn about psychosocial risk factors, listen to the employees, and take relevant action to handle the issues. The leaders received 2 types of training at different levels: (1) PSC training, and (2) job design workshops. For the PSC training, leaders were introduced to PSC theory and principles, and they reflected on the PSC of their own teams. A discussion was also conducted with a focus on areas for improvement and appropriate action plans. Throughout the training, the leaders were expected to learn how to identify, target, and reduce potential psychosocial risks and work on improving, promoting, and strengthening a favorable climate (ie, PSC). As an example, in Table 2 we have provided a detailed description of how a PSC workshop was conducted.

**Table 2:** Training example.

**Psychosocial Safety Climate (PSC) workshop** PSC is a corporate climate that benefits employees’ psychological health and well-being. Beyond this, PSC is related to productivity, sick leave and workers’ compensation claims. To improve PSC in an organization, the leaders could be trained to address PSC.^11^ Below is an example of the workshop content: 1) What is PSC—Introduction to psychosocial risks factors at work and PSC, including: • psychosocial risk factors at work • definitions of PSC • the 4 elements of PSC • the theoretical framework of PSC. 2) Why PSC is important—Evidence of PSC, including: • the functions of PSC as a leading indicator • the functions of PSC as a moderator • the link between PSC and health, motivation, and productivity. 3) How to measure PSC—PSC scale and the benchmarks^19^^,^^20^, including: • the PSC-12 • a PSC risk level and prognosis. 4) How to build PSC—Introduction of the intervention principle, involving the: • PSC hierarchy of control (PSC-HOC) framework.^21^ 5) Action planning and follow-up, which involves: • identifying problems in teams • discussing and brainstorming actions • focusing on achievable actions • performing follow-up meetings, if possible • adjusting and updating action plans.

A PSC intervention should also include employees’ and other stakeholders’ participation and involvement in the decision-making processes^6^ and allow them to hear each other’s perspective. Including employees’ voice in an intervention process enables actual issues they have experienced to be tackled and the process to be refined to match the needs of employees.^6^ Involving employees and other parties also increases the ownership of the intervention, and thus more people engage in facilitating change.^15^ Employees can be involved in an intervention via many approaches, such as by having safety representatives in a steering group during action planning or by collecting employees’ perspectives through surveys and interviews during risk assessment processes. An example of activities conducted in the organization was encouraging employees to verbalize their concerns about work stress and psychological health. Opening lines of communication is important to close the gap between employers’ perceptions and employees’ experiences.

Organizational communication is equally important. When policies and procedures are changed, for example, the implementation of a new workload policy, the message should be communicated to all levels of employees to ensure that they fully understand the change. Without effective communication, discrepancies in perception can occur between the employers and employees. Similarly, employers and employees whose perceptions and experiences are not aligned represent a significant hurdle and could even cause an intervention to fail. In Case Study 1, information about well-being and psychological health and support was constantly communicated to employees. Videos, online courses, and events were used to convey the importance of psychological health, the company’s services available to protect employees’ well-being, and examples of how other workers were managing their well-being issues.

#### 2.1.3. Measurements and results of PSC

The 12-item PSC-12 scale was used to assess PSC.^19^ An example item is “In my organization, the prevention of stress involves all levels of the organization.” The PSC scores range from 12 to 60, with higher scores indicating better PSC. Items are scored on a 5-point Likert scale (1 = Strongly disagree to 5 = Strongly agree). The Cronbach α of this scale was α = .95 at T1, α = .95 at T2, and α = .96 at T3.

From 2018 to 2021, the organization’s PSC improved significantly (*F*[2] = 68.42, *P* < .001; Table 3). In 2018, the organization scored 39.35 (SD = 10.12) on their PSC, indicating a medium-risk situation (Table 4). In 2019, the organization achieved a high PSC (low-risk situation) with a PSC level of 40.80 (SD = 9.16) (*t*-value = 2.74, *df* = 1334, *P* < .001). In 2021, we observed further improvement in their PSC level, recorded at 44.07 (SD = 9.57), indicating a low-risk situation.^20^ (For the scale of PSC-12, scores range from 12 to 60, which according to Bailey et al,^20^ indicate specific risk levels: PSC ≤ 26 = very high-risk; 26 < PSC ≤ 37 = high-risk; 37 < PSC < 41 = medium-risk; and PSC ≥ 41 = low-risk.) A summary independent *t* test suggested that PSC levels significantly improved from 2019 to 2021 (*t*-value = 7.68, *df* = 2780, *P* < .001). (We applied a Bonferroni correction for multiple comparisons, in which the *P* threshold for the test would be *P* < .025. The results are still positive.)

**Table 3:** Mean and SD of psychosocial safety climate (PSC) across the years for Case Studies 1 and 2.

	**Case Study 1**					
	**Time 1 (*n* = 690)**		**Time 2 (*n* = 646)**		**Time 3 (*n* = 2136)**	
	**Mean**	**SD**	**Mean**	**SD**	**Mean**	**SD**
**PSC**	39.35	10.12	40.80	9.16	44.07	9.57

	**Case Study 2**					
			**Time 1 (*n* = 1333)**		**Time 2 (*n* = 1707)**	
			**Mean**	**SD**	**Mean**	**SD**
		**PSC**	46.36	8.01	46.15	8.45

**Table 4:** Distribution of respondents at different risk levels for Case Study 1.

**PSC risk level**	**Range 12-60**	**2018**	**2019**	**2021**
**High PSC**	≥41	347 (46.9%)	337 (52.2%)	1440 (67.4%)
**Medium PSC**	41< and >37	89 (12%)	78 (12.1%)	200 (9.4%)
**Low PSC**	37≤ and >26	217 (29.3%)	189 (29.3%)	388 (18.2%)
**Very low PSC**	≤26	87 (11.8%)	42 (6.5%)	108 (5.1%)

Abbreviation: PSC, psychosocial safety climate.

Adapted from Bailey et al.^20^

### 2.2. Case Study 2

#### 2.2.1. Background and context

Case Study 2 involved a private international company with nearly 5000 employees. Online surveys were distributed in February 2021 and February 2022, with safety and health personnel collecting the survey data via an online platform. The study information and survey link were emailed directly from the organization’s safety and health personnel to all employees. A password-encrypted deidentified dataset was sent to the researchers. In total, 1350 responses were collected from the survey in 2021 (T1) and 1707 responses in 2022 (T2). At T1, 49% of respondents were female, median age group was 30-39 years old, and 95% were working full time. At T2, 48.4% of respondents were female, median age group was 30-39 years old, and 89.9% were full-time employees.

#### 2.2.2. Intervention

Several interventions were performed in the organization from February 2021 to February 2022, which were initiated by the organization’s workplace champion (ie, the mental health officer). A mental health officer was recruited at the end of 2020 to oversee the psychological health of the organization’s workers (Table 5). A risk assessment was then conducted to gauge the company’s PSC. In response to the risk assessment, training was provided to leaders, who were trained about psychosocial risks, and to teams with comparatively lower PSC, who additionally received an action plan workshop. Employee representatives were also recruited to help initiate well-being programs in teams. Some of the changes that were made in the organization included introducing work-from-home policies and providing additional sick (mental health) leave and rebranding all sick leave as well-being leave to reduce stigma (Table 1). This change is directly linked to the organizational priority of psychological health over productivity. The priorities of an organization are revealed in their daily operations, activities, and processes. Organizational policies they implemented, such as encouraging employees to take annual leave and well-being leave and developing efficient psychological injury claims processes, imply an emphasis on the importance of psychological health.

**Table 5:** Example of a well-being initiative*.*

**Appointing a mental health officer** Over these years, many corporates have recruited a specialist in health and well-being and given them the corporate title of “mental health officer.” Unlike traditional safety and health personnel, who focus solely on physical safety, the mental health officer encompasses a duty of care concerning workers’ psychological well-being. Organization X in Case Study 2 followed this trend. In 2020, Organization X introduced a new corporate role for workers’ well-being with a “head of well-being” job title. The head of well-being is responsible for designing, planning, and implementing strategies for building a psychologically healthier workplace. The roles of a mental health officer are broad but highly specific. They are broad because a mental health officer has many tasks, which share a specific goal—protecting and promoting workers’ well-being. To be a mental health officer is challenging. They must maintain high performance, productivity, and business growth while also supporting and protecting workers from overwork, stress, and exhaustion. It was necessary to learn how to manage the role more effectively. An initial step taken by the head of well-being in Organization X was to engage with scholars to measure psychosocial safety climate in their organization. Mental health officers received adequate support through collaboration, knowledge sharing, empirical evidence, expert recommendations, and idea exchanges. A collaboration between academics and practitioners also generates new ideas and future research opportunities. Although it is a legal obligation to take care of workers’ psychological health, appointing a specific well-being role is a step beyond legal obligations.

Similarly to Case Study 1, the researchers participated in an ongoing discussion with the organization’s representative to keep track of the planned and performed activities. Following the PSC assessment at T1, the organization actively focused on mental health and preventing distress and provided support particularly to teams with low PSC to help them improve PSC. We categorized the performed activities into different levels of intervention, and in doing so observed that intervention occurred at all levels of the organization. Unlike Case Study 1, even though PSC had also been introduced, no PSC-specific workshops or training were provided to leaders. Instead, leaders were trained in general psychosocial risk factors at work, such as understanding job demands and job resources.

#### 2.2.3. Measurements and PSC results

Measures similar to those used in Case Study 1 were used. Cronbach α was .93 at T1 and .94 at T2 (Table 3). Ethics approval was obtained from the authors’ university (ethics protocol: 203691). At T1, the organizational PSC score was 46.38 (SD = 8.01). In 2022, the PSC score was equivalent at 46.15 (SD = 8.45), with an independent *t* test indicating no significant changes in PSC scores from T1 and T2 (*t*-value = 0.70, *df* = 3038, *P* > .05). At different risk levels, we found there was a reduction in the high-PSC category from 76.7% in year 2021 to 73.8% in year 2022, and more employees reported lower levels of PSC, or higher risk categories (Table 6).

**Table 6:** The distribution of respondents at different risk levels for Case Study 2.

**PSC risk level**	**Range 12-60**	**2021**	**2022**
**High PSC**	≥41	1022 (76.7%)	1260 (73.8%)
**Medium PSC**	41< and >37	143 (10.7%)	185 (10.8%)
**Low PSC**	37≤ and >26	145 (10.9%)	225 (13.2%)
**Very low PSC**	≤26	23 (1.7%)	37 (2.2%)

## 3. Discussion

We found that levels of PSC improved with an intervention that involved multiple levels of organization (organization, organization-employee interface, employee) and focused on the 4 PSC elements, including management priority of psychological health before productivity, management commitment to and support of psychological health, communication on psychological health-related issues, and participation and involvement in psychological health matters.

By considering both the mean and dispersion, we found PSC improvements in Case Study 1, in which the PSC increased significantly from T1 to T2 and then continually improved from T2 to T3. Moreover, we observed that fewer individuals in the organization reported a very low level of PSC (reduced from 11.8% to 5.1%). Our findings suggest that to ensure effective interventions, organizations should focus on the issues at each level of influence and perform appropriate training and activities at each level. Although organizational PSC did not improve over time in Case Study 2, the organization remained at a high PSC level. It should be noted that in Case Study 2 in 2021, 76.7% of personnel reported high PSC—this is the highest level of PSC we have seen in Australia. Improvements at this high level may be more difficult to attain than in Case Study 1, which at a lower PSC had greater room for improvement. A sizable organizational expansion and restructuring between T1 and T2 in Case Study 2 should also be noted, as well as a high organizational voluntary turnover (this coincided with the post-COVID phenomenon of “the great resignation”). It is likely that the high PSC level created a platform of dynamic stability^22^^,^^23^ within which challenges and major disruption could be managed.

In both Case Studies 1 and 2 we observed that the intervention involved all levels of the organization. At the organizational level, new policies and risk assessment to identify problems with current psychosocial risks were introduced. These new policies were communicated to lower-level employees, which is an integral aspect of PSC. In addition to organizational-level interventions, leaders received workshops and training to understand psychosocial risks and possible actions to tackle them. Their practices regarding existing policies and PSC practices should increase individual perceptions of PSC by improving the work context, and also provide a context within which the effects of any tertiary intervention are optimized. Tertiary interventions such as employee assistance programs can help employees manage distress, and activities and events such as awareness events and psychoeducation can help employees better understand the risks of psychosocial factors. Overall, we found that PSC can be significantly improved by integrating efforts at all organization levels, by first starting with the organizational system and working downwards to individual-level factors. Organizational efforts and activities for promoting psychological health, revising system policies and practices, and providing training and resources reflect a strong system emphasis on individual psychological health, which was evident in both Case Studies 1 and 2.

Analysis of each activity and program indicated that each PSC element was included. Additionally, both case studies revealed that efforts required to improve or build a psychologically healthy workplace should start with management motivation and priorities,^3^ which will stimulate initiatives required to prevent work stress. For example, initiatives that introduce new policies should be supported by *management commitment*, be *communicated* to all organization members, and be regularly revised by *consulting and involving all organization stakeholders*.

Case Studies 1 and 2 aimed to identify the gaps in the organizations’ current policies and procedures regarding psychological health-related issues by using risk assessment. Conducting a risk assessment also reflects the organization’s readiness to learn about its weaknesses, its capacity to make adjustments and improvements in how it manages psychosocial risk factors, and its ability to demonstrate that it prioritizes the psychological health of its employees. In Case Study 2, the organization created a new role of mental health officer (Table 2). Having a specialist to focus on workers’ psychological health reflects the organization’s willingness to improve it. It also reflects the organization’s readiness to enter a new beginning by incorporating the psychological aspect of well-being into its strategic plans. Taking action and making changes to psychosocial risk policies at the organizational level provides the primary ingredient for building PSC. The change in policies can be further enacted into daily practices and operations by the middle-level management and communicated to employees.

Both cases conducted training for their leaders (even though in Case Study 2, leaders were only trained on job demands and resources but not specifically about PSC) illustrating a commitment to management and support for tackling psychosocial risk issues. Leaders were trained to understand psychosocial risks, especially PSC. In a workshop, leaders were trained to comprehend the 4 PSC principles, generating discussion on potential actions to improve these categories. A PSC capacity-building workshop prepares the leaders on the fundamental ideals of PSC and its principles. The leaders’ PSC training had at least 2 functions: to improve PSC over time and to sustain PSC function despite external challenges, such as the global pandemic.^4^ In our Case Study 1, the assessment of PSC had been delayed during the pandemic in 2020. Although previous evidence has shown that such an external major event would initiate conversations related to mental health^11^ and hence improve PSC, not all events are expected to have similar effects, for example, economic recession and world conflicts. These elements were expected to affect the organization’s PSC negatively. Therefore, we suggest that the intervention may have demonstrated a protective effect in terms of sustaining and protecting workers from internal and external challenges. Although PSC training sometimes did not occur, instead a basic psychosocial risks workshop was conducted (ie, in Case Study 2). This type of intervention focuses on job redesign, which requires an enactment of the principles of PSC.

In Case Study 1, the organization communicated with employees through the internal staff website, highlighting changes made to existing organizational policies, procedures, and systems. The staff portal was also used to provide ample resources to help leaders manage their team members. Another way of enhancing better communication was by using positive language. In Case Study 2, “sick leave” was renamed “well-being leave” to convey that employees were free to take leave for mental health concerns and extra leave was allowed. In addition to that they introduced lifestyle leave. Additionally, timely updates from leaders were likely helpful in keeping employees informed about the organization’s strategies and goals. Blogs were uploaded in Case Study 2 to communicate the organization’s recent activities and information about psychological health services, such as counseling. Through effective communication, organizations could send a strong message about their priorities for psychological health protection for workers, which helps build high-level PSC.

In both cases, employee participation and involvement was encouraged in the intervention decision-making processes. During the risk assessment process, employee perceptions were collected through the PSC survey to understand how they viewed their organizational climate. Further, safety representatives were also recruited to speak for the employees, to help organize and implement a psychosocial risk promotion program, and to communicate with leaders about their needs. Critically, their involvement created a social dialogue between the employers and employees. Social dialogue is recognized as an important pillar to build a decent workplace. It allows the employees to democratically participate and influence psychosocial issues. Social dialogue in the organizational structure is necessary for developing, implementing, and sustaining a work stress intervention. Social dialogue ultimately provides opportunities for employees, especially those at the lowest level and those with the least power to voice their concerns.

### 3.1. Other factors that could influence the results

Acknowledging that workplace interventions are multifactorial, different factors could interfere with the results for the cases. Considering the time gap, some studies have shown that PSC can improve within a year and there is a need to consistently work to keep the PSC level high. In a recent study, Berglund and colleagues^24^ found that the level of PSC improved within 6 months and was sustained during a 12-month follow-up period. We used a 1-year time gap in the current study as a recurring annual check time for PSC assessment and to provide adequate time for an organizational-level change. Different from an intervention that is often designed and carried out in teams,^11^^,^^24^ our case studies surveyed the phenomenon at a broader scope with a whole of organization approach capturing the complexity of organizational dynamics.

Organization size arguably is a factor influencing the transmission of organizational values and priorities from the top echelon to the lower level of entities. Sizable organizations require more time and systematic strategies in ensuring employees at all levels are informed, are involved, and participate in the intervention process, while at the same time ensuring that their voice is heard and transmitted up to the top entities. In this current study, effective communication was used to transfer messages about the process of assessment, activities, and introducing new policies; this was evident in an improvement in the PSC communication subscale, particularly in Case Study 1 (mean = 9.52, SD = 2.72, at T1; mean = 10.40, SD = 2.30, at T2; mean = 11.08, SD = 2.39, at T3).

### 3.2. Theoretical and practical implications

From a theoretical perspective, the current study provides an extended understanding of intervention theories and advances the PSC literature. Whereas the work stress intervention literature often focuses on the task-level job design, such as reducing demands and improving resources as implied in the job demands-resources theory, or individual-level interventions such as mindfulness and stress management interventions, our study combined the contemporary work stress intervention literature and the PSC elements (which is the leading indicator of job design and occupational health and safety outcomes), with results implying that by incorporating the 4 elements of PSC and conducting activities at all levels (organization, organization-person, person) PSC can be built within the workplace.

The construct of PSC consists of the 4 important elements or principles that capture the fundamental ideas of a successful process for managing psychosocial risks and with a priority on psychological health and well-being. PSC in its theoretical framework embodies the best principles or enablers of an organization intervention that might focus on a range of levels of intervention. We suggest that practicing the principles of PSC is exercising the system, which can build the PSC while targeting working conditions and individual behaviors. It is by regularly exercising these principles^9^ that organizations can get better at managing, regulating policies, and designing programs that are pro-worker, which is evident in the current article. We also have seen the importance of organizational commitment from the top management team in investing and initiating change within organizations.

Our research informs the question about what works best in improving PSC. Applying the principles of PSC builds the PSC. In this study after providing training, advice, and consultations, we gave complete autonomy to organizational members to implement any strategy that they thought would improve PSC, using the PSC principles. In this way, members had complete autonomy over the “what,” “where,” “when,” and “who” aspects of the intervention (of course there may have been organizational constraints). A useful analogy is surfing. You can train a surfer about skills and techniques, but on the water with all the dynamics that water waves bring, a surfer must read the situation and adjust strategy. Practice makes perfect. In theorizing what works in PSC interventions we can say that multilevel interventions, with autonomy over content, can lead to improvement in organizational PSC.

Practically, this study presents several possible ways of planning and conducting programs, activities, and events to improve organizational PSC. We contribute to the knowledge of applying the system approach of work stress intervention and PSC principles for the development of sustainable and participatory interventions. Both cases have detailed how organizational internal experts can integrate the elements of PSC into their existing policies and practices, therein amending and rectifying their risk management system. An example is the training of leaders in how to enact PSC in their day-to-day practices. Workshops conducted by experts and/or regular meetings among leaders or with safety representatives could help leaders to explore and develop their capacity in dealing with different psychosocial risks. The case studies also showed that to protect the organization from external turbulent and economic challenges, implementing PSC-based interventions can help protect workers from these situations.

### 3.3. Limitations and future research recommendations

First, neither case study was designed with the suggested gold standard of a randomized controlled trial (RCT). However, RCTs require highly controlled conditions that are not likely viable in real-world settings and raise the ethical concern of allocating workers to a no intervention support condition (ie, control) resulting in an unequal distribution of the research benefits. RCTs also assume that the context of the intervention will be constant, which is not always the case in real-life scenarios (eg, COVID turbulence), thus threatening ecological validity.

Second, no overall evaluation of the effectiveness of specific intervention components was conducted. The intervention conducted in the selected period was vast, and it was difficult to tailor the best program or specific intervention that most effectively boosts PSC over time. Although the PSC evaluation was on an organizational level, some individuals might experience more benefits from the intervention by attending the training or by having a leader who attended the training. In contrast to most of the existing literature on intervention, our intervention was not limited to a specific workshop, content, or structure. The interventions illustrated here capture all levels of activities, programs, and actions in tackling and managing psychosocial risks at work.

Evaluating PSC is evaluating the intervention process. As argued previously, PSC itself is comprised of process elements, and evaluating change in PSC provides intelligence about how well the system functions and how well the coordinate system of interventions guided by PSC principles is working. The before and after evaluations of PSC are of great interest to the organization in terms of organizational learning. Whereas work stress interventions are usually evaluated in terms of process, to answer the main questions such as “were best practice principles used?” and “was the intervention implemented?” our research focused on whether best practice principles were implemented (ie, assessed by PSC) but we gave great latitude in how to get there (which makes it difficult to pinpoint what specific aspect worked).

Future studies could consider measuring other elements of the intervention process, such as the readiness of organizations and the effectiveness, and whether interventions claimed to be implemented were in fact implemented. Early planning would be required to ensure a well-designed evaluation process and an effective post hoc evaluation of the intervention that promotes understanding the fidelity of the implemented activities and program.

In addition, we would recommend a real-time monitoring (RTM) system to capture the day-to-day PSC enactment at workplaces. An RTM system would provide timely and instant feedback to the managers or supervisors on the status of their workplace psychosocial safety and prompt action to correct the issues and problems without delay. Research by McLinton et al^25^ showed daily changes in a PSC pulse survey and highlights the applicability and effectiveness of RTM in capturing day-to-day PSC enactment. Although the concept of PSC is arguably relatively stable, it could fluctuate around the mean level or general state of PSC due to daily happenings and events, which can provide information more specifically on how supervisors or leaders manage the occurrence of psychosocial risk events.

## 4. Conclusion

We reviewed 2 intervention case studies to provide insights about how an organization can improve PSC by engaging in various types of system changes and programs. The case studies suggested that an integrated approach for intervention involving the social coordination of organizational-level, organization-employee interface, and employee-level interventions effectively promotes a psychosocially safe climate. We also offer insights into how to bridge research and practice. By collaborating with academics and practitioners, organizations can implement empirical, evidence-based practice to improve PSC. Efforts must focus on building a strong and positive climate through a collective social coordination process.

## Data Availability

The data underlying this article were provided by third parties (ie, the participatory organizations) under a mutual research agreement and cannot be shared.
